# The Role of Apoptosis in the Cellular Response of Liver and Spleen of BALB/c Mice in Cutaneous Leishmaniasis

**Published:** 2015-03

**Authors:** Mahvash Jafari, Shanaz Shirbazou, Seyed Homayoon Sadraie, Gholamreza Kaka, Majid Norozi

**Affiliations:** 1 Molecular Biology Research Center, Baqiyatallah University of Medical Sciences, Tehran, Iran;; 2 Department of Parasitology, Faculty of Medicine, Baqiyatallah University of Medical Sciences, Tehran, Iran;; 3 Neuroscience Research Center, Faculty of Medicine, Baqiyatallah University of Medical Sciences, Tehran, Iran;; 4 Department of Biochemistry, Faculty of Medicine, Baqiyatallah University of Medical Sciences, Tehran, Iran

**Keywords:** Cutaneous leishmaniasis, Apoptosis, Inbred BALB C mice, Liver, Spleen

## Abstract

**Background:**

Cutaneous leishmaniasis is a common parasitic disease in Iran being mainly caused by *Leishmania (L.) major*. The aim of this study was to investigate the occurrence of apoptosis in the spleen and liver of female mice infected with* L. major*.

**Methods:**

BALB/c mice were randomly assigned into the control and experimental groups (ten mice per group). The experimental groups were subcutaneously inoculated with promastigotes of *L. major* at stationary phase. The animals were sacrificed after 20, 40, 60, 90, and 120 days of injection. The liver and spleen were analyzed for various parameters of apoptosis.

**Results:**

Activities of superoxide dismutase and caspase-3, levels of superoxide anion production and malondialdehyde, and the percent of DNA fragmentation were increased in the liver and spleen of the infected mice. Catalase activity in the liver was increased, while glutathione level in both tissues was decreased after 90 and 120 days of infection. The numbers of apoptotic nuclei in the spleen were higher than the liver at 90 and 120 days post-infection using the TUNEL method.

**Conclusion:**

*L. major* infection induces a time-dependent increase in apoptosis in the liver and spleen as evidenced by the production of ROS, increasing activation of caspase-3, elevated DNA fragmentation, and increasing lipid peroxidation. Induction of oxidative stress was observed in the liver and spleen after 90 and 120 days of initiation of the infection. However, the spleen tissue appears to be more sensitive to the infection to* L. major* on oxidative stress and apoptosis induction compared with the liver tissue.

## Introduction

Leishmaniasis is an infectious disease caused by over 20 different species of the protozoan parasite *Leishmania (L)*. It is a major public health problem in the world that affects over 12 million people in 88 countries, 350 million people at risk, and almost 2 million new cases per year. Each year leishmaniasis kills 60,000 people. Leishmaniasis manifests itself as a mild cutaneous lesion, mucocutaneous disease, or fatal visceral form depending upon the species of the parasites.* Leishmania* (*L.*)* major* is the principal agent of cutaneous leishmaniasis (CL) in Iran. The annual incidence of new cases of CL is around 1.5 million.^1^^,^^2^* Leishmania* parasites live as flagellated promastigotes in the gut of the insect vector and as non-flagellated amastigotes in the vertebrate host macrophages.^3^

The infected cells counteract the invasive pathogen by programmed cell death or apoptosis. Apoptosis is an important mechanism known to regulate cell populations in tissues and organs. Apoptosis, as a death process, plays a major role in the removal of infected, mutated, or damaged cells during development, tissue homeostasis, and aging of multicellular organisms. Apoptosis associates with distinctive morphological and biochemical changes, including cytochrome c release, caspase-family proteases activation, nuclear chromatin condensation, and DNA fragmentation. Apoptosis plays an important role in a number of diseases, including heart disease and cancer.^4^^,^^5^

Understanding the molecular and cellular pathways activated in response to infection that leads to cell death is important for controlling the parasitic diseases.^5^ It is therefore necessary to investigate apoptosis in parasite and host in vivo and their correlation during *Leishmania* infection. An apoptosis-like cell death has been observed in *Leishmania* promastigotes and amastigotes.^6^^,^^7^ Apoptosis can be induced by a variety of agents such as drugs or hydrogen peroxide or nitric oxide donors.^6^^,^^8^^,^^9^ There are several reports showing that apoptosis occur in macrophages and T host cells infected with leishmaniasis.^10^^,^^11^ However, there is no study on the time-dependent effects of *L. major* infection on the induction of apoptosis in tissues. In the present work, we evaluate the occurrence of apoptosis in the spleen and liver of BALB/c mice infected with* L. major*. BALB/c mice were chosen due to their high sensitivity to *L. major *infection.^11^

## Materials and Methods

*Chemicals*

All chemicals used were of extra pure grade and obtained from Sigma and Merck. RPMI 1640 medium and fetal calf serum (FCS) were from Gibco (UK). 

*Animals*

Female BALB/c mice (20-30 g, 6-8 weeks of age) were obtained from Pastour Institute (Tehran, Iran) and acclimated for at least two weeks prior to the experimental use. All animals were fed with standard mouse chow and water *ad libitum* and maintained under standard conditions at 20-22°C temperature and 60±10% relative humidity with a 12 h light/dark cycle. All procedures were in accordance with the standards for animal care established by the Ethical Committee of the Baqiyatallah University of Medical Sciences.

*Parasite Culture*

MRHO/IR/75/ER of *L. major* as a prevalent strain of CL in Iran was maintained in BALB/c mice. Amostigotes were isolated from mice spleens, and then transformed to promastigotes in Novy-Nicolle-Mac Neal (NNN) medium supplemented with penicillin (100 U/ml), streptomycin (100 µg/ml) and 20% heat-inactivated FCS at 22±1°C. Subsequently, the third passage promastigotes from NNN medium were progressively adapted to RPMI 1640 media supplemented with antibiotics, L-glutamine (30 mg/L) and FCS.^12^

*Mice Infections*

BALB/c mice were randomly divided into control and experimental groups, ten mice in each group. Control groups received RPMI 1640 medium. Experimental groups were subcutaneously inoculated in the base of the tail with 2×10^6^ promastigotes of *L. major* at stationary phase in 0.1 ml culture medium.^13^ Mortality of the animals was recorded up to 120 days after inoculation.

*Tissue Preparation*

At 20, 40, 60, 90 and 120 days after inoculation, seven mice in each group were scarified under ether anesthesia and the liver and spleen were quickly removed and washed in ice-cold phosphate buffer saline (PBS). Washed tissues were immediately immersed in liquid nitrogen and stored at -70°C until biochemical analysis.

On the day of use, frozen tissue samples were quickly weighed and homogenized 1:10 in ice-cold PBS. The homogenates were then centrifuged at 16,000 g for 15 min at 4°C. The supernatants were separated and used for enzyme activities assays and protein determination. Liver and spleen tissues were fixed in 0.01 M phosphate-buffered (pH 7.4) 10% formalin and embedded in paraffin for histopathological sectioning.

*Superoxide Dismutase (SOD) Activity Assay*

The activity of SOD was determined according to Paoletti and Mocali.^14^ For an assay, the samples were read on a Genesys 10 UV spectrophotometer at 340 nm for 5 min by the oxidation of NADH. One unit of activity is defined as the amount of enzyme that inhibits the oxidation of NADH by 50% at 25°C and the results were expressed as U/mg protein.

*Catalase (CAT) Activity Assay*

CAT activity in tissue homogenates was measured spectrophotometrically at 240 nm by calculating the rate of degradation of H_2_O_2_, the substrate of the enzyme using the method of Aebi.^15^ Specific activity is expressed as 1 µmole H_2_O_2_ decomposed min^-1^mg^-1^ protein.

*Determination of Glutathione (GSH) Level*

GSH level was determined by the method of Tietz.^16^ Cellular protein was precipitated by the addition of 5% sulfosalicylic acid and removed by centrifugation at 2000 g for 10 min. GSH in the supernatant was assayed at 412 nm by monitoring the absorbance of 5, 5’-dithiobis 2-nitrobenzoic acid (DTNB) for 5 min. The level of GSH was expressed as nmol/mg protein.

*Determination of Malondialdehyde (MDA) Level*

Liver and spleen MDA level as a marker of lipid peroxidation was determined at 532 nm using 2-thiobarbituric acid according to the method of Ohkawa.^17^ MDA concentration was expressed as nmol/mg protein.

*Protein Level Assay*

The total protein concentrations in the cytosols were measured by Branford^’^s method using bovine serum albumin as standard.^18^

*Assay for Superoxide Anion Production Level*

Tissue homogenates were centrifuged at 2000 g for 15 min at 4°C. Superoxide anion production level in the packed cells was determined from the absorbance reading at 550 nm and expressed as nmol/mg protein.^19^

*Detection of DNA Fragmentation*

*Quantitative Analysis of DNA Fragmentation*

Quantitation of DNA fragmentation was determined as described by Burton.^20^ A small piece (<1 cm^3^) of tissues were quickly weighed and powdered in the liquid nitrogen. The proportion of fragmented DNA in the powdered tissues was calculated from the absorbance reading at 600 nm by colorimetric diphenylamine assay.

*Analysis of DNA on Agarose Gel*

DNA from mice liver and spleen was prepared according to the procedure of Garner.^21^ A small piece (<1 cm^3^) of frozen tissue samples were quickly weighed and powdered in the liquid nitrogen. DNA from the powdered tissues was extracted by phenol/chloroform/isoamylalcohol method. Then, the DNA samples were dissolved in TBE buffer (90 mM Tris–HCl, 2 mM EDTA and 90 mM boric acid pH 8) and electrophoresis was performed on a 1.2% agarose gel. The gel was stained in ethidium bromide and visualized in UV light. EcoR1-Hind III digested DNA was used as a DNA size marker.

*In Situ Labeling of DNA Fragments*

*In situ* detection of DNA fragments, in uninfected and infected liver and spleen sections at 90 and 120 days, was performed by Terminal deoxynucleotidyltransferase enzyme (TdT)-mediated dUTP nick-end labeling (TUNEL) using a Cell Death Detection kit (ApopTag, Japan) according to manufacturer’s instructions. Samples were counterstained with 0.5% methyl green prior to analysis by light microscope. Apoptotic nuclei appeared dark brown.

*Caspase-3 Activity Assay*

Caspase-3 protease activity was measured using Fluorometric Immunosorbent Enzyme Assay (FIENA) kit from Roche Applied Science. Endpoint fluorescence was measured at λ_ex_=400 nm and λ_em_=550 nm.

*Statistical Analysis*

All calculations were performed using INSTAT statistical software. For the time-dependent studies, the data were statistically analyzed using variance (ANOVA) analysis followed by Tukey post hoc multiple comparison test. P values less than 0.05 were considered statistically significant. Results were expressed as means±SD, with n denoting the number of experiments performed. 

## Results

*The Mortality of Mice*

No mortality occurred in the group of mice receiving *L. major* 20 days after inoculation. Mortality was observed at 40 (2%), 60 (2%) and 70-90 (23.53%) days post-infection and no more death was seen up to 120 days after the inoculation. Mice in experimental groups showed redness and swelling of the inoculated basal tail on the 8th to 12th day post-infection which increased progressively with time. The wound was detected due to parasite growth after 4-6 weeks at the injection site, which increased in size with time.

*GSH and MDA Levels*

Mean liver and spleen GSH and MDA levels in the control and experimental groups at different post-infection times are depicted in table 1. MDA level was increased 5.72, 9.15 and 14.05% (P=0.03) in the liver and 9.41, 16.46 (P=0.03) and 22.27% (P=0.003) in the spleen at 60, 90, and 120 days post-infection. GSH level was decreased 5.97, 12.62 (P=0.03) and 16.88% (P=0.003) in the liver and 11.53 (P=0.04), 14.71 (P=0.006) and 21.81% (P=0.0006) in the spleen at 60, 90, and 120 days post-infection.

**Table 1:** Effect of *L. major* infection on liver and spleen GSH and MDA levels in control and experimental mice at different times

**Time (day)**	**GSH (nmol/mg protein)**		**MDA (nmol/mg protein)**	
	**Liver**	**Spleen**	**Liver**	**Spleen**
Control	101.23±6.75	59.24±4.45	6.12±0.45	7.23±0.62
20	100.98±8.32	57.09±3.87	6.24±0.36	7.41±0.57
40	98.59±6.59	55.73±3.14	6.36±0.42	7.66±0.54
60	95.19±5.25	52.41±3.48^*^	6.47±0.39	7.91±0.65
90	88.46±6.12^*,#^	50.53±3.36^**,#^	6.68±0.55	8.42±0.57^*^
120	84.14±5.89^**,#^	46.32±3.71^***,#^	6.98±0.45^*^	8.84±0.74^**,#^

Values are expressed as mean±SD (n=7). Control is mean of the control groups at different time intervals. ^*^P=0.03 and ^**^P=0.003 for liver GSH, ^*^P=0.04, ^**^P=0.006 and ^***^P=0.0006 for spleen GSH, ^*^P=0.03 for liver MDA, ^*^P=0.03 and ^**^P=0.003 for spleen MDA vs. control. ^#^P=0.04 vs. other times in infected mice.

*Antioxidant Enzyme Activities*

**Figure 1:**
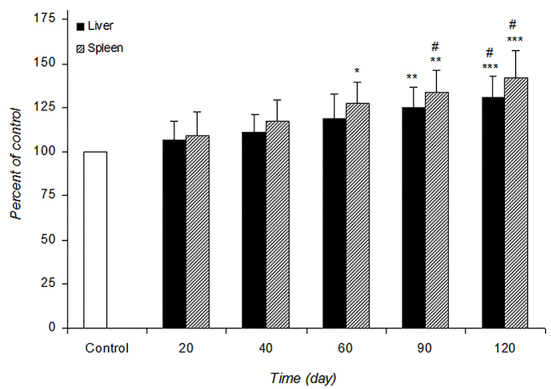
The alteration of SOD activity in control and infected mice during the experimental period. Values are expressed as percentage of control (100%)±SD (n=7). Control is mean of the control groups at different time intervals. **P=0.008 and ***P=0.0009 for liver SOD and *P=0.03, **P=0.004 and ***P=0.0008 for spleen SOD vs. control. #P=0.03 vs. other times in infected mice.

**Figure 2:**
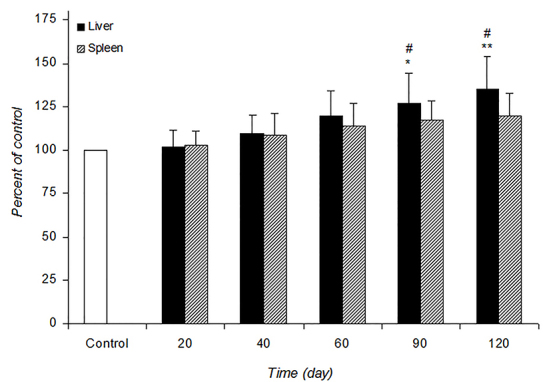
The alteration of CAT activity in control and infected mice at different times during the experimental period. Values are expressed as percentage of control (100%)±SD (n=7). Control is mean of the control groups at different time intervals. *P=0.03 and **P=0.005 vs. control. #P=0.03 vs. other times in infected mice.

Figures 1 and 2 shows the alteration of SOD and CAT activities in the control and infected mice at different post-infection times. SOD activity was significantly increased in the liver at 90 (P=0.008) and 120 days (P=0.0009) and the spleen at 60 (P=0.03), 90 (P=0.004) and 120 (P=0.0008) days of infected mice compared with the control. The increased CAT activity in the liver was observed at 90 (P=0.03) and 120 (P=0.005) days post-infection. *L. major* infection did not cause any noticeable increase in the spleen CAT activity at different post-infection times.

*Superoxide Anion Production Level*

The effect of *L. major* infection on the liver and spleen superoxide anion production level in the control and experimental mice is summarized in table 2. Superoxide anion production level was significantly increased 13.57, 18.96 (P=0.02) and 23.55% (P=0.003) in the liver and 19.38 (P=0.03), 26.48 (P=0.003) and 35.46% (P=0.0006) in the spleen at 60, 90, and 120 days post-infection.

**Table 2:** Effect of *L. major* infection on liver and spleen superoxide anion production level and caspase-3 activity in control and experimental mice at different times

**Time (day)**	** O_2_^●-^ (nmol/mg protein) **		**Caspase-3 activity (nmol/mg protein)**	
	**Liver**	**Spleen**	**Liver**	**Spleen**
Control	0.501±0.045	0.423±0.045	51.027±0.622	57.531±0.666
20	0.519±0.032	0.445±0.035	51.126±0.451	57.869±0.511
40	0.542±0.038	0.462±0.037	51.471±0.728	58.154±0.516
60	0.569±0.044	0.505±0.049^*^	51.885±0.765	58.467±0.657
90	0.596±0.057^*^	0.535±0.045^**,#^	52.013±1.098	58.794±0.718^*^
120	0.619±0.051^**,#^	0.573±0.044^***,#^	52.185±0.486^*^	58.965±0.815^**^

Values are expressed as mean±SD (n=7). Control is mean of the control groups at different time intervals. ^*^P=0.02 and ^**^P=0.003 for liver O_2_^●-^, ^*^P=0.03, ^**^P=0.003 and ^***^P=0.0006 for spleen O_2_^●-^, ^*^P=0.03 for liver caspase-3, ^*^P=0.03 and ^**^P=0.008 for spleen caspase-3 vs. control. ^#^P=0.04 vs. other times in infected mice.

*Caspase-3 Activity*

Table 2 shows the alteration of caspase-3 activity in the control and experimental groups at different times. Caspase-3 activity was significantly increased in the liver (at 120 days, P=0.03) and the spleen (at 90 days with P=0.03 and 120 days with P=0.008) of the infected mice compared with the control.

*Percent of DNA Fragmentation*

**Figure 3:**
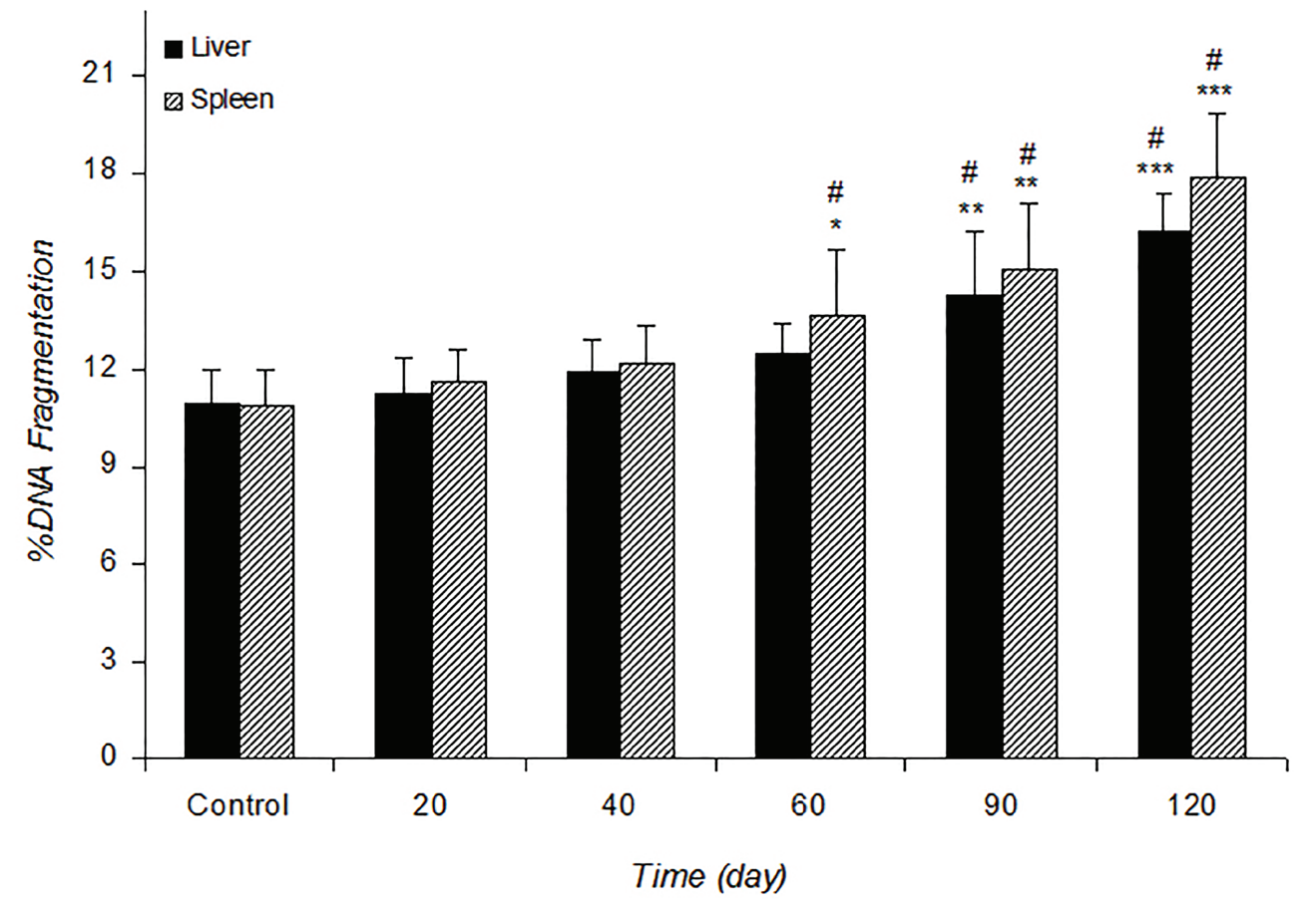
Effect of L. major infection on liver and spleen DNA fragmentation percent in control and experimental mice at different times. Values are expressed as mean±SD (n=7). Control is mean of the control groups at different time intervals. **P=0.004 and ***P=0.0004 for liver and *P=0.05, **P=0.003 and ***P=0.0003 for spleen %DNA fragmentation vs. control. #P=0.03 vs. other times in infected mice.

**Figure 4:**
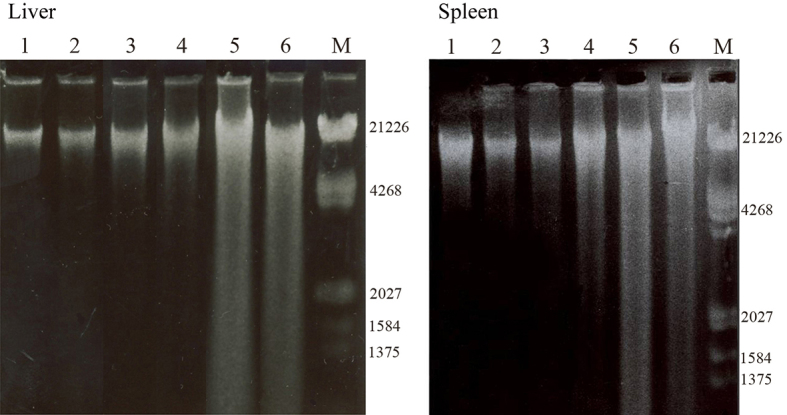
1.2% agarose gel electrophoresis of liver and spleen DNA infected with L. major during the experimental period. Lanes 1-6 are control, 20, 40, 60, 90 and 120 days post-infection, respectively. M is EcoR1-Hind III standard DNA

Quantitative DNA fragmentation analysis in the liver and spleen of infected mice was determined by diphenylamine procedure as presented in figure 3. The DNA fragmentation showed time-dependent effects of *L. major* infection. Percent of DNA fragmentation significantly increased in the liver (at 90, P=0.004 and 120 P=0.0004 days) and the spleen (at 60, P=0.05, 90, P=0.003 and 120, P=0.0003 days) of infected mice compared with the control groups. Agarose gel electrophoresis of genomic DNA isolated from the treated and the control mice is given in figure 4.* L. major* infection did not induce any significant fragmentation of cellular DNA at 20, 40 days (for both tissues) and 60 days (for liver) post-infection (lanes 2-4) and the patterns were similar to that of the control (lane 1). Extensive DNA degradation as smears was observed in treated cells at higher times (lane 4 for spleen, lanes 5 and 6 for both tissues), which was in agreement with diphenylamine analysis of DNA fragmentation as described above.

**Figure 5:**
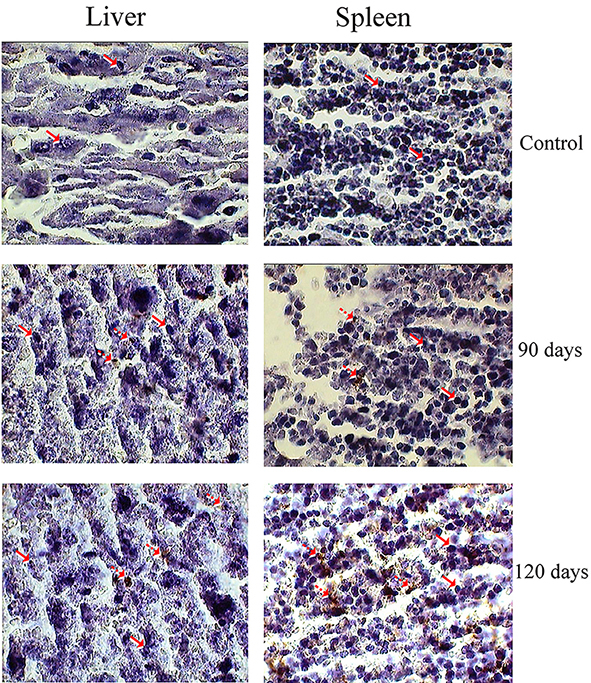
Photomicrographs of TUNEL staining on representative liver and spleen sections infected with L. major at 90 and 120 days. Arrows indicate intact cells and interrupted arrows indicate TUNEL-positive cells (400× magnification).

Quantitation of apoptosis in the liver and spleen cells based on TdT labeling indices is depicted in figure 5. It shows that higher TUNEL-positive staining in the liver and spleen cells in treated groups at 90 and 120 days compared with the control groups. A large number of apoptotic nuclei were observed in both cells at 120 days post-infection*.* As shown in table 3, the percentage of apoptotic cells increased with increasing infection time. Only 1-2.5% of the cells were stained in control, whereas 7.37 (P=0.009) and 12.89% (P=0.0006) of cells stained in the liver and spleen at 120 days post-infection. The percentage of apoptotic cells in the spleen was higher than liver at 120 days post-infection (P=0.03).

**Table 3:** Percent of apoptotic cells in control and infected mice at 90 and 120 days post-infection

**Time (day)**	**Apoptotic cells (%)**	
	**Liver**	**Spleen**
Control	1.28±0.98	2.49±1.28
90	4.86±3.65	5.68±2.87^#^
120	7.37±3.32^**^	12.89±4.86^***,†^

Values are expressed as mean±SD (n=7). Control is mean of the control groups at different time intervals. ^**^P=0.009 and ^***^P=0.0006 vs. control, ^#^P=0.04 vs. 120 days in infected spleen mice and ^†^P=0.03 vs. 120 days in infected mice liver.

## Discussion

The molecular mechanisms that lead to induction or inhibition of apoptosis in *Leishmania *and host cells are important for the development of anti-*Leishmania *medications.^22^ Induction and inhibition of apoptosis of macrophages and dendritic cell infected with *Leishmania *were reported in several studies.^7^^,^^10^^,^^23^

Infection of BALB/c mice to *L. major* is associated with the activation of Th2 cells secreting cytokines.^2^^,^^11^ Cytokines activated macrophages release a great number of reactive oxygen species (ROS), which are responsible for parasite killing in macrophages. ROS can cause extensive damage to cellular proteins, lipids, and DNA.^24^ Antioxidative enzymes such as SOD and CAT form the first line of defence against ROS in the organism. SOD is a vital enzyme that detoxiﬁes superoxide to hydrogen peroxide and CAT convert hydrogen peroxide to H_2_O.^3^^,^^25^ In this study, superoxide anion production level and SOD activity in the liver (>90 days) and spleen (>60 days) and CAT activity in the liver (>90 days) were significantly increased in infected mice. The increased SOD and CAT activities in the liver are due to the compensatory up-regulation of these antioxidants after the initial generation of ROS.^26^ The increased SOD activity without change in CAT activity in the spleen leads to the accumulation of H_2_O_2_ in this tissue, which may be the cause of the induction of oxidative stress.^27^ Oxidative stress, as a signaling event, is shown to trigger programmed cell death (apoptosis). This pattern is similar to the obtained results from mouse skin tissue infected with *L. *major.^28^ Several studies reported a reduction of CAT and SOD activities of erythrocytes in hamsters and humans and a significant decrease in serum total antioxidant status in dog’s liver and kidney infected with *L. infantum*.^29^^-^^31^ A study demonstrated that superoxide anion production was significantly elevated during phagocytosis of the stationary phase promastigotes.^10^

Glutathione (GSH) participates in many vital cellular processes that include storage and transport of cysteine, cell proliferation, and regulation of apoptosis. It acts as a free radical scavenger and as a substrate for several enzymes, including glutathione peroxidase and glutathione-S-transferase.^32^ A significant depletion of GSH was noted in the present study in a time dependent manner in mice liver (>60 days) and spleen (>40 days) tissues infected with *L. major* that is due to high oxidative stress and over utilization of GSH by cells. Depletion of GSH leads to oxidized GSH (GSSG) production and finally decreased GSH/GSSG ratio in tissues of infected mice, which is an index of tissue oxidative stress.^26^ Our finding is in agreement with the results of previous reports that infection with parasites depleted GSH in host erythrocytes and whole blood.^29^^,^^33^^,^^34^ However, the increased GSH level in erythrocytes of CL patients was shown by other studies.^34^^,^^35^

Lipid peroxidation (LPO) is caused by oxidative modification due to the presence of ROS. A common marker of LPO is MDA, which has been frequently used as markers of oxidative stress in response to different agent such as infection.^26^^,^^34^ The present study showed that MDA level was significantly increased in the liver (at 120 days) and spleen (>60 days) in infected mice. The enhanced LPO shows that *Leishmania* infection-induced ROS are not totally scavenged by the antioxidant enzymes in both tissues. Increased level of erythrocytes MDA have been described in visceral leishmaniasis (VL) in Hamsters,^27^^,^^29^ dogs,^33^ and humans.^34^ Heidarpour et al. showed a significant increase in the serum MDA level observed in dog liver and kidney infected with *L. infantum*.^31^ Serarslan et al. showed that LPO level of patients with active CL was significantly higher.^35^

The family of cysteine proteases, called caspases, plays a critical role during apoptosis. Several different caspases are constitutively expressed in cells and the most prevalent caspase in the cell is caspase-3. This caspase is ultimately responsible for the initiation of DNA fragmentation and morphologic events.^5^^,^^6^ Significant increase of caspase-3 activity at the end of 90 (for both tissues) and 120 days (for spleen) of infection in the present investigation corresponds with the induction of apoptosis. In several studies, extensive evidence for the existence of caspase-like activities associated with parasite apoptosis has been published.^36^^,^^37^

ROS can damage DNA bases that lead to strand breaks and chromatin cross-linking. Fragmentation of DNA is one of the hallmarks of apoptotic cell death.^6^ To identify DNA fragmentation in the liver and spleen by *L. major* infection, two different methods, including diphenylamine method and agarose gel electrophoresis were used in this study. Both methods clearly showed infection-induced DNA fragmentation in both tissues after 90 and 120 days. In addition, DNA fragmentation patterns on agarose gel similar to what is seen in apoptotic cells is obtained; suggesting that *L. major* infection at 90 and 120 days induce apoptosis. Similar results were observed in the skin and lung of mice infected with *L.* *major.^38^*Kocyigit et al. showed increased DNA damage and oxidative stress in patients with CL.^39^ In addition, DNA fragmentation during induction of apoptosis has been reported by a variety of stress conditions in *Leishmania*.^5^^,^^9^^,^^22^

In this study, the TUNEL method was used to further investigate apoptosis characteristics, which was able to demonstrate a number of liver and spleen with 3-OH DNA ends generated.^7^^,^^36^ The quantification of the number of nuclei observed by this method showed that the percentage of apoptotic cells were increased with increasing infection time and the peak of enhancement was observed at 120 days in both cells. However, the number of apoptotic nuclei in the spleen was higher than the liver (figure 4 and table 3). Lindoso and co-workers reported that apoptosis in amastigotes from hamsters infected with visceral leishmaniasis appeared 90-day post-infection in the liver and spleen, as analyzed using the TUNEL method. Necrosis was not present in these tissues.^7^

In this study, *L. major* infection in the liver and spleen induces oxidative stress through the generation of ROS, depletion of GSH, increased lipid peroxidation and DNA damage, which may lead to increase of intracellular Ca^2+^ level and cell death.^1^^,^^5^ High concentrations of ROS and RNS have pro-apoptotic effects and prevent the development of parasite.^2^^,^^5^^,^^11^ The presence of apoptosis has also been reported in *leishmania *and host cells of other unicellular organisms.^9^^,^^11^In addition, several reports show that antileishmanial drugs induce apoptosis in *Leishmania*.^22^^,^^40^ The production of viable parasites reduces the initiation of apoptosis in parasite-positive host cells.^6^ However, additional studies will be needed to better understand the effects of *L. major* infection on induction of cell death in a variety of cell types using an *in vivo* system.

## Conclusion

The current study suggests that *L. major* infection induces a time-dependent increase in apoptosis in both tissues as evidenced by the production of ROS, activation of caspase-3 and DNA fragmentation. In addition, oxidative stress induction was observed in the liver and spleen after 90 and 120 days of infection initiation. The elevated DNA fragmentation may be related to increased oxidative stress. However, spleen tissue appears to be more sensitive to the infection to *L. major* on induction of oxidative stress and apoptosis compared to liver tissue.
